# Amphotericin B resistance in *Leishmania amazonensis*: *In vitro* and *in vivo* characterization of a Brazilian clinical isolate

**DOI:** 10.1371/journal.pntd.0012175

**Published:** 2024-05-20

**Authors:** Bianca A. Ferreira, Elizabeth M. Coser, Stephane de la Roca, Juliana I. Aoki, Nilson Branco, Gustavo H. C. Soares, Mayara I. S. Lima, Adriano C. Coelho

**Affiliations:** 1 Departamento de Biologia Animal, Instituto de Biologia, Universidade Estadual de Campinas (UNICAMP), Campinas, Brazil; 2 Departamento de Parasitologia, Instituto de Ciências Biomédicas, Universidade de São Paulo, São Paulo, Brazil; 3 Departamento de Biologia, Programas de Pós Graduação em Saúde e Ambiente e Ciências da Saúde, Universidade Federal do Maranhão, São Luís, Brazil; Universidade Federal de Minas Gerais, BRAZIL

## Abstract

In Brazil, *Leishmania amazonensis* is the etiological agent of cutaneous and diffuse cutaneous leishmaniasis. The state of Maranhão in the Northeast of Brazil is prevalent for these clinical forms of the disease and also has high rates of HIV infection. Here, we characterized the drug susceptibility of a *L*. *amazonensis* clinical isolate from a 46-year-old man with diffuse cutaneous leishmaniasis coinfected with HIV from this endemic area. This patient underwent several therapeutic regimens with meglumine antimoniate, liposomal amphotericin B, and pentamidine, without success. *In vitro* susceptibility assays against promastigotes and intracellular amastigotes demonstrated that this isolate had low susceptibility to amphotericin B, when compared with the reference strain of this species that is considered susceptible to antileishmanial drugs. Additionally, we investigated whether the low *in vitro* susceptibility would affect the *in vivo* response to amphotericin B treatment. The drug was effective in reducing the lesion size and parasite burden in mice infected with the reference strain, whereas those infected with the clinical isolate and a resistant line (generated experimentally by stepwise selection) were refractory to amphotericin B treatment. To evaluate whether the isolate was intrinsically resistant to amphotericin B in animals, infected mice were treated with other drugs that had not been used in the treatment of the patient (miltefosine, paromomycin, and a combination of both). Our findings demonstrated that all drug schemes were able to reduce lesion size and parasite burden in animals infected with the clinical isolate, confirming the amphotericin B-resistance phenotype. These findings indicate that the treatment failure observed in the patient may be associated with amphotericin B resistance, and demonstrate the potential emergence of amphotericin B-resistant *L*. *amazonensis* isolates in an area of Brazil endemic for cutaneous leishmaniasis.

## Author summary

The parasitic protozoan *Leishmania amazonensis* is the etiological agent responsible for cutaneous and diffuse cutaneous leishmaniasis. The latter is characterized by nodular lesions that spread over the body of the infected individual and is associated with a CD4/Th2 type immune response to the infection. Treatment failure is commonly reported for this clinical form of disease. Here, we characterized a *L*. *amazonensis* clinical isolate obtained from a patient with diffuse cutaneous leishmaniasis coinfected with HIV who had undergone unsuccessful therapeutic regimens with meglumine antimoniate, liposomal amphotericin B, and pentamidine. To evaluate whether the treatment failure was due to drug resistance in this clinical isolate, *in vitro* susceptibility assays were performed with the antileishmanial drugs currently available, followed by an investigation of treatment response in mice. *In vivo* studies demonstrated that mice infected with this clinical isolate did not respond to treatment with amphotericin B, but did respond to treatment with miltefosine, paromomycin, and a combination of both drugs, confirming an amphotericin B resistance phenotype of the isolate that was also observed *in vitro*.

## Introduction

Leishmaniasis is a complex of diseases caused by the protozoan parasite of the genus *Leishmania*. This complex comprises two distinct manifestations of the disease: visceral (VL) and tegumentary leishmaniasis (TL). In Brazil, seven species of *Leishmania* are responsible for TL, the clinical presentations of which can be further classified as localized cutaneous (LCL), disseminated, mucocutaneous, or diffuse cutaneous leishmaniasis (DCL) [1]. Among the endemic species in Brazil, *Leishmania amazonensis* is one of the causative species of LCL, a clinical form characterized by a single or small number of lesions that develop as a papula at the site of an infected sand fly bite and ulcerate slowly over time [1]. This species is also associated with DCL in patients with impairment of the T cell response to *Leishmania* antigens [2]. In Latin America, more than 1 million cases of TL were reported from 2001 to 2021, and 37% of the cases occurred in Brazil [3]. Although the number of cases in Brazil reduced by 14.3% from 2017 to 2021, more than 15,000 cases were reported in 2021, occurring in almost all Brazilian states, mainly in the Amazon region [3]. The main risk factors associated with the disease are male sex, young people (up to 15 years old), poor quality of houses, and living close to forested areas [1].

The treatment for leishmaniasis is limited to a few drugs, such as pentavalent antimonials (SbV), which have remained as the first choice for several decades in some endemic areas, including Brazil, despite their low efficacy rates [4]. Miltefosine (MF), an oral drug, is an alternative drug that has been approved for TL treatment in Brazil since 2018, with cure rates of approximately 70% [5,6]. Paromomycin (PM), an aminoglycoside antibiotic, was proposed as an alternative option for TL treatment as a topical agent [7] and for the treatment of VL in Southeast Asia and East Africa, via the parenteral route, as a monotherapy or in combination with MF or amphotericin B (AmB) [8–11]. AmB has two formulations that require intravenous administration: deoxycholate and liposomal AmB (L-AmB). The first is highly toxic, while L-AmB has lower toxicity; both formulations have similar rates of efficacy for TL (>80%) [4,12]. The therapeutic regimen of this drug depends on the formulation, and ranges from 1 to 5 mg/kg/day over 20 to 30 consecutive days [13,14]. AmB has been used for the treatment of leishmaniasis in Brazil for cases of therapeutic failure and patients coinfected with human immunodeficiency virus (HIV). Most VL cases that have reported AmB treatment failure were associated with the immune status of the host, such as HIV coinfection or another immunocompromised state not directly related to the parasite [15–17], while a limited number of studies have confirmed clinical resistance of VL patients to AmB [18–20]. On the other hand, no reports of clinical resistance associated with TL currently exist in the literature.

The main target of AmB in *Leishmania* is ergosterol, the major membrane sterol of the parasite that the drug interacts with high avidity, forming pores in the plasma membrane, changing the permeability to ions and metabolites, and generating reactive oxygen species [21–23]. AmB-resistant lines experimentally selected *in vitro* have already been reported for different species of the parasite [24,25]. Resistant parasites exhibit, as the main mechanism of resistance, changes in sterol composition that are linked to mutations in genes involved in sterol biosynthesis, such as those that encode sterol C-24-methyltransferase, sterol C-14α-demethylase, and sterol C-5 desaturase [24,26–28].

Recently, the case of a 46-year-old patient living in the state of Maranhão, northeast Brazil, with DCL, caused by *L*. *amazonensis*, and HIV coinfection was reported [29]. This patient underwent therapeutic regimens with L-AmB, SbV, and pentamidine (PEN), which were followed by relapses. The parasite responsible for the disease in this patient, which was refractory to treatment with two of the main drugs used against leishmaniasis in Brazil (SbV [Glucantime] and L-AmB), was isolated. In this study, we characterized the *in vitro* and *in vivo* susceptibility to AmB, and a resistance phenotype was found for this clinical isolate when compared with the susceptibility of a susceptible reference strain and an AmB-resistant line selected *in vitro*. The *in vivo* resistance phenotype was confirmed by evaluating the effectiveness of MF and PM as a monotherapy or in combination in animals infected with this clinical isolate. This study describes the first report of a *L*. *amazonensis* clinical isolate resistant to AmB, and indicates the emergence of AmB-resistant parasites in an endemic area for leishmaniasis in Brazil.

## Materials and methods

### Ethics statement

For experiments using mice, protocols and procedures were approved by the Ethics Committee for Animal Experimentation of the Instituto de Biologia, Universidade Estadual de Campinas (UNICAMP) (protocols: 5571–1/2020 and 5719-1/2021).

### Drugs

For the *in vitro* susceptibility assays, stock solutions of MF (Sigma-Aldrich; 100 mM), SbIII (Sigma-Aldrich; 100 mM), SbV (Glucantime, Sanofi-Aventis; 100 mM), and PEN (Sigma-Aldrich; 10 mM) were diluted in Milli-Q ultrapure water, filter-sterilized (0.22 μm pore size), and then kept at -20°C until use. AmB deoxycholate (Sigma-Aldrich; 1 mM) was diluted in DMSO (Sigma-Aldrich). For *in vivo* assays, AmB deoxycholate (Cristália), MF, and PM were diluted in phosphate-buffered saline (PBS) and kept in stock solutions at 5 mg/mL, 3 mg/mL, and 60 mg/mL respectively.

### Parasite cultivation and selection of an AmB-resistant line

The clinical isolate AAB (MHOM/BR/2019/AAB-MA) was previously isolated from a DCL patient coinfected with HIV from Maranhãozinho, Maranhão, Brazil and typed as *L*. *amazonensis* [29]. This isolate was registered in SisGen (*Sistema Nacional de Gestão do Patrimônio Genético e do Conhecimento Tradicional Associado*—Brazil) under the accession number ADA06AF. Promastigotes of *L*. *amazonensis* reference strain (MHOM/BR/1973/M2269) and the AAB clinical isolate were grown at 25°C in M199 medium (Sigma-Aldrich) supplemented with 40 mM HEPES (pH 7.4), 0.1 mM adenine, 5 μg/mL hemin, 10% heat-inactivated fetal bovine serum, 100 U/mL penicillin, and 100 μg/mL streptomycin [30].

An AmB-resistant line was selected by exposing promastigotes of the M2269 strain to increasing AmB concentrations, starting at 25 nM until achieving a final concentration of 200 nM. Clonal lines were obtained from the AmB-resistant population (AmB200) after plating onto M199 medium containing 1% agar (Thermo Fisher Scientific). After 10–15 days, colonies were picked and expanded in liquid M199 medium containing AmB.

### Drug susceptibility assays against promastigotes and intracellular amastigotes of *L*. *amazonensis*

The susceptibility of antileishmanial drugs against promastigotes of the M2269 strain, the AAB clinical isolate, and an AmB-resistant clonal line was evaluated using the MTT (3-(4,5-dimethylthiazol-2-yl)-2,5-diphenyltetrazoline bromide) colorimetric assay, as previously described [31]. Briefly, 2×10^6^ log-phase parasites were incubated in presence of the following drugs serially diluted (1:2): AmB (800 to 3.12 nM), SbIII (1,000 to 1.56 μM), PEN (100 to 1.56 μM), MF (200 to 3.12 μM), or PM (1,000 to 6.25 μM) for 24 h. Three independent experiments were performed in triplicate and the 50% effective concentration (EC_50_) was determined by sigmoidal regression curves generated using the GraphPad Prism 8 software.

The drug susceptibility assays for intracellular amastigotes were performed as previously described [31]. Briefly, bone marrow-derived macrophages (BMDMs) obtained from BALB/c mice were incubated in a 5% CO_2_ atmosphere at 37°C [32]. Macrophages were infected with stationary-phase promastigotes of the M2269 strain, AAB isolate, and AmB-resistant line at a ratio of 5:1 (parasites:macrophage) and incubated at 34°C in a 5% CO_2_ atmosphere. Non-internalized parasites were removed after 3–4 h by washing with warmed PBS. Next, infected macrophages were treated with the drugs serially diluted at the following concentration ranges: AmB (150 to 0.78 nM), PEN (0.4 to 0.01 μM), MF (20 to 0.31 μM), or PM (500 to 1 μM) for 72 h and SbV (1,000 to 25 μM) for 144 h. The percentage of infection and number of amastigotes per infected macrophage were determined by counting at least 100 macrophages in three independent experiments that were used to determine EC_50_ values as described above.

### Antileishmanial drug treatment in mice infected with *L*. *amazonensis*

Female BALB/c mice (4–6 weeks) were obtained from the *Centro Multidisciplinar para Investigação Biológica* (CEMIB) of UNICAMP and kept in mini-isolators, receiving food and water *ad libitum*. For evaluation of AmB effectiveness, female BALB/c mice were randomly grouped and infected with either the M2269 strain, AAB isolate, or AmB-resistant line by inoculating 1×10^6^ stationary-phase promastigotes resuspended in 30 μL of filter-sterilized PBS into the right hind footpad. Animals were treated with 15 doses of AmB, starting at the 4^th^ week post-infection, with doses of 1, 5, or 10 mg/kg/day administered intraperitoneally. Similarly, *in vivo* experiments were performed to evaluate the effectiveness of MF, PM, and MF plus PM in mice infected with the M2269 strain or the AAB isolate. The dosages used were 15 mg/kg/day of MF by oral route (gavage), 600 mg/kg/day of PM by intraperitoneal route, or 8 mg/kg/day of MF plus 300 mg/kg/day of PM for 15 days as previously described [33,34]. For all *in vivo* experiments, an untreated group infected with each parasite line was used as a control.

Lesion size was measured weekly with a caliper (Mitutoyo Corporation, Japan) and, at the end of the treatment, lesion tissues of each animal were submitted to parasite burden quantification through quantitative real-time PCR, as previously described [34]. Histopathological examination was also conducted with isolated infected hind footpad fragments that were fixed with formalin and then processed with paraffin. Sections were stained with hematoxylin-eosin and then visualized under an optical microscope. Finally, to assess the toxicity of AmB, the body weight of the animals was recorded before and post-treatment, and the levels of aspartate transaminase (AST), alanine transaminase (ALT), and creatinine in the serum of untreated and treated animals were measured at the end of the treatment period, as previously described [34].

### Statistical analysis

Statistical analyses of the data were performed using the GraphPad Prism 8 software by applying the one-way ANOVA test and Tukey’s multiple comparison post-test; *p* values <0.05 were considered statistically significant.

## Results

### *In vitro* drug susceptibility and phenotypic characterization of the *L*. *amazonensis* AAB clinical isolate

**Fig 1:**
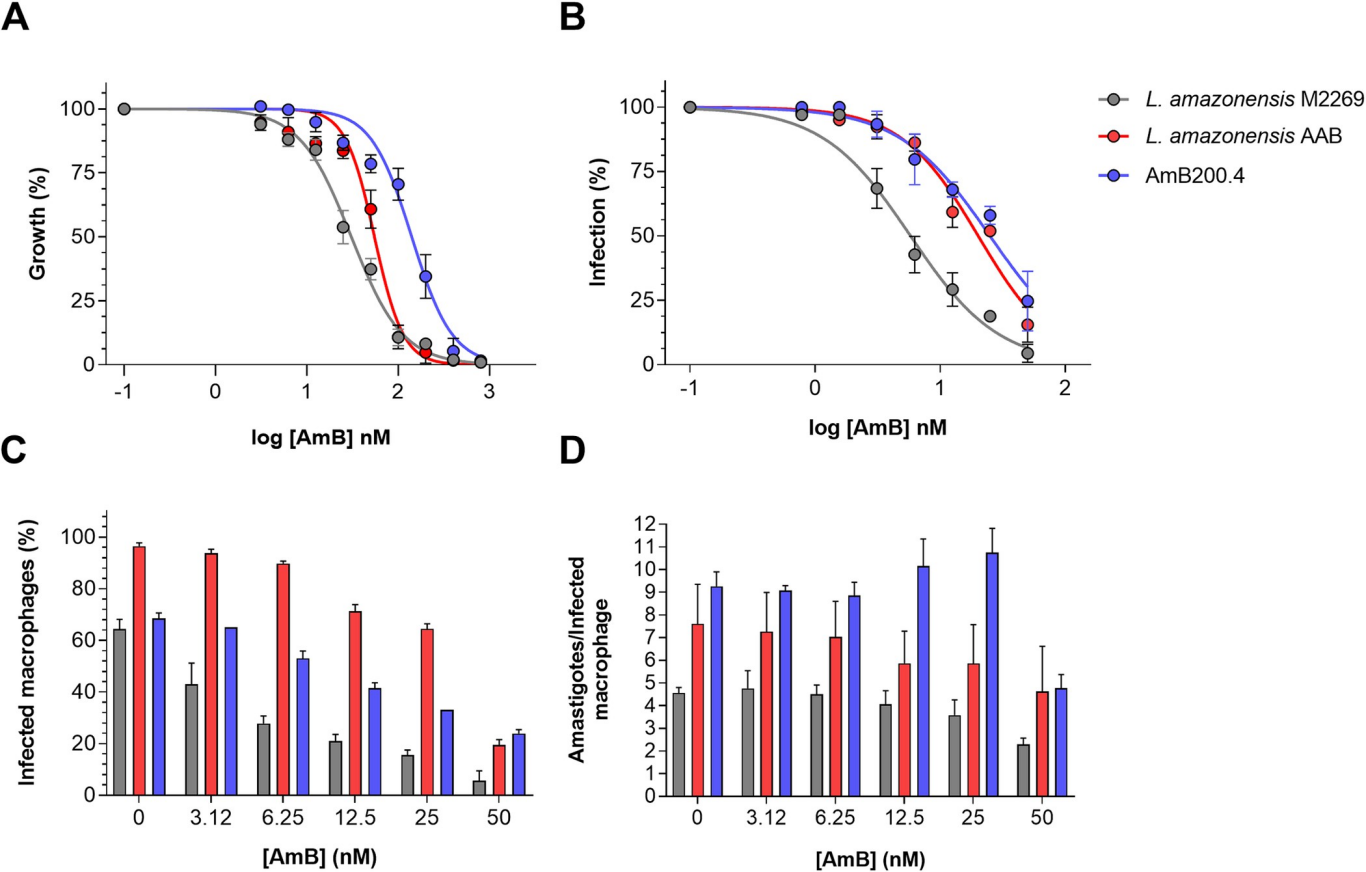
*In vitro* activity of AmB against promastigotes and amastigotes of *L*. *amazonensis* M2269 strain, the AAB clinical isolate, and the AmB-resistant clonal line (AmB200.4). (A) Promastigotes were exposed to increasing concentrations of AmB for 24 h and viability was determined by the MTT assay. (B) Bone marrow-derived macrophages (BMDMs) were infected with stationary-phase promastigotes and exposed to increasing concentrations of AmB for 72 h. (C) Percentage of infected BMDMs and (D) number of intracellular amastigotes per infected macrophage following treatment with the indicated concentrations of AmB. The average ± standard deviation of three independent experiments is shown.

The AAB clinical isolate previously typed as *L*. *amazonensis* was obtained from a patient exposed to therapeutic regimens with L-AmB, SbV, and PEN [29]. To investigate whether the therapeutic failure observed in the patient was related to a resistance phenotype of the AAB isolate, *in vitro* drug susceptibility tests were performed in parallel with the *L*. *amazonensis* M2269 strain [35], considered as reference by the World Health Organization and susceptible to all drugs used in the chemotherapy of leishmaniasis. Previous reports have shown that *L*. *amazonensis* clinical isolates from Brazil are uniformly sensitive to AmB [36,37]. In the present study, the EC_50_ values of AmB were 2- and 3.5-fold higher for the AAB isolate than for the M2269 strain in the promastigote and amastigote stages, respectively (Table 1 and Fig 1). Therefore, we focused on investigating the potential AmB resistance phenotype in the AAB isolate.

**Table 1:** Activity of antileishmanial drugs against promastigotes and intracellular amastigotes of *L*. *amazonensis* M2269 reference strain, the AAB clinical isolate, and an AmB-resistant clonal line (AmB200.4).

Drugs	Promastigotes ^c^			Intracellular Amastigotes ^c^			CC_50_ ^d^
	M2269	AAB	AmB200.4	M2269	AAB	AmB200.4	
**AmB** ^a^	29.02 ± 4.46	57.25 ± 4.38	124.73 ± 3.78	5.88 ± 0.78	20.88 ± 2.35	24.09 ± 5.35	127.36 ± 0.94
**SbIII** ^b^	30.53 ± 5.71	33.59 ± 4.85	7.56 ± 2.25	-	-	-	-
**SbV** ^b^	-	-	-	511.23 ± 11.4	>1,000	194.3 ± 14.31	>2,000
**PEN** ^b^	2.98 ± 0.44	1.42 ± 0.39	2.83 ± 0.83	0.2 ± 0.05	0.27 ± 0.07	0.13 ± 0.05	0.36 ± 0.02
**MF** ^b^	17.55 ± 1.41	23.1 ± 0.16	44.32 ± 2.12	2.04 ± 0.23	9.16 ± 0.92	7.5 ± 0.92	49.52 ± 2.93
**PM** ^b^	145.23 ± 23.04	147.53 ± 18.58	163.9 ± 21.55	53.56 ± 2.38	125.16 ± 4.5	40.37 ± 6.6	536.60 ± 27.1

^a^ Concentrations in nM. AmB, amphotericin B

^b^ Concentrations in μM. SbIII, trivalent antimony; SbV, pentavalent antimony; PEN, pentamidine; MF, miltefosine; and PM, paromomycin

^c^ EC_50_ mean values ± standard deviation of three independent experiments

^d^ 50% cytotoxic concentration (CC_50_) of each drug that reduces bone marrow-derived macrophage viability by 50%, as determined previously [31]

(-) Data not determined.

To evaluate the resistance phenotype in the AAB isolate, we obtained AmB-resistant parasites through stepwise selection of the M2269 strain in the promastigote stage. The selection was started with an initial concentration of 25 nM of AmB (equivalent to the EC_50_ of this strain) (Table 1), until a final concentration of 200 nM was reached, after approximately 90 days in culture (S1 Fig). The AmB-resistant population, AmB200, had an EC_50_ of AmB that was 6.3-fold higher than that of the M2269 strain (S1 Table). Four independent clones from the AmB200 population (AmB200.1, AmB200.2, AmB200.3, and AmB200.4) were obtained and evaluated for AmB susceptibility, which had EC_50_ values that ranged from 81 to 124.73 nM, i.e., 2.8 to 4.3-fold higher than that of the parental M2269 strain (S1 Table). We chose the AmB200.4 clone for further characterization. In the promastigote stage, the AmB200.4 clonal line had a lower growth rate until the early stationary phase when compared to the growth curves of the M2269 strain and the AAB isolate, which exhibited similar rates of growth over 7 days in culture (S2 Fig). In the absence of drug, the AmB200.4 line and the M2269 strain had similar rates of infection in BMDMs (64.4 ± 3.8% and 68.5 ± 2.1%, respectively); while the average number of amastigotes per macrophage was higher for the AmB200.4 line (9.36 ± 0.64) than for the M2269 strain (4.56 ± 0.24) (Fig 1C and 1D). The rate of infection in BMDMs was higher for the AAB isolate (96.5 ± 1.29%) compared to those for the AmB200.4 line and the M2269 strain, while the average number of amastigotes per macrophage (7.61 ± 1.75) was higher than for the M2269 strain (Fig 1C and 1D). Similar to the AAB isolate, the AmB200.4 line was also considered resistant to AmB in the amastigote stage, with an EC_50_ that was 4.1-fold higher than that for the M2269 strain (Table 1 and Fig 1B–1D).

Susceptibility assays were also carried out for the other drugs used in the chemotherapy of leishmaniasis. When compared with the results for the M2269 strain, the AAB isolate in the amastigote stage was considered resistant to SbV, since the EC_50_ was at least 2-fold higher than that for the M2269 strain; despite no significant differences in the EC_50_ values for the clinical isolate and the reference strain in the promastigote stage (Table 1). For PEN, both strains presented similar EC_50_ values in both stages, indicating that the AAB isolate is susceptible to this drug (Table 1). Additionally, we evaluated MF and PM susceptibility, two drugs that had not been used in the treatment of the patient, and the clinical isolate was found to be less susceptible to both drugs when compared with the results for the M2269 strain in the amastigote stage, with EC_50_ values that were 2.4 and 4.5-fold higher, respectively. As promastigotes, both the M2269 strain and the AAB isolate were similarly susceptible to these drugs (Table 1). Finally, the AmB200.4 line showed cross resistance to MF in both stages of the parasite, while this line was highly susceptible to antimonials (SbIII and SbV) when compared to the parental M2269 strain (Table 1).

### *In vivo* treatment with AmB of mice infected with the *L*. *amazonensis* AAB clinical isolate, the M2269 reference strain, and the AmB200.4 line

**Fig 2:**
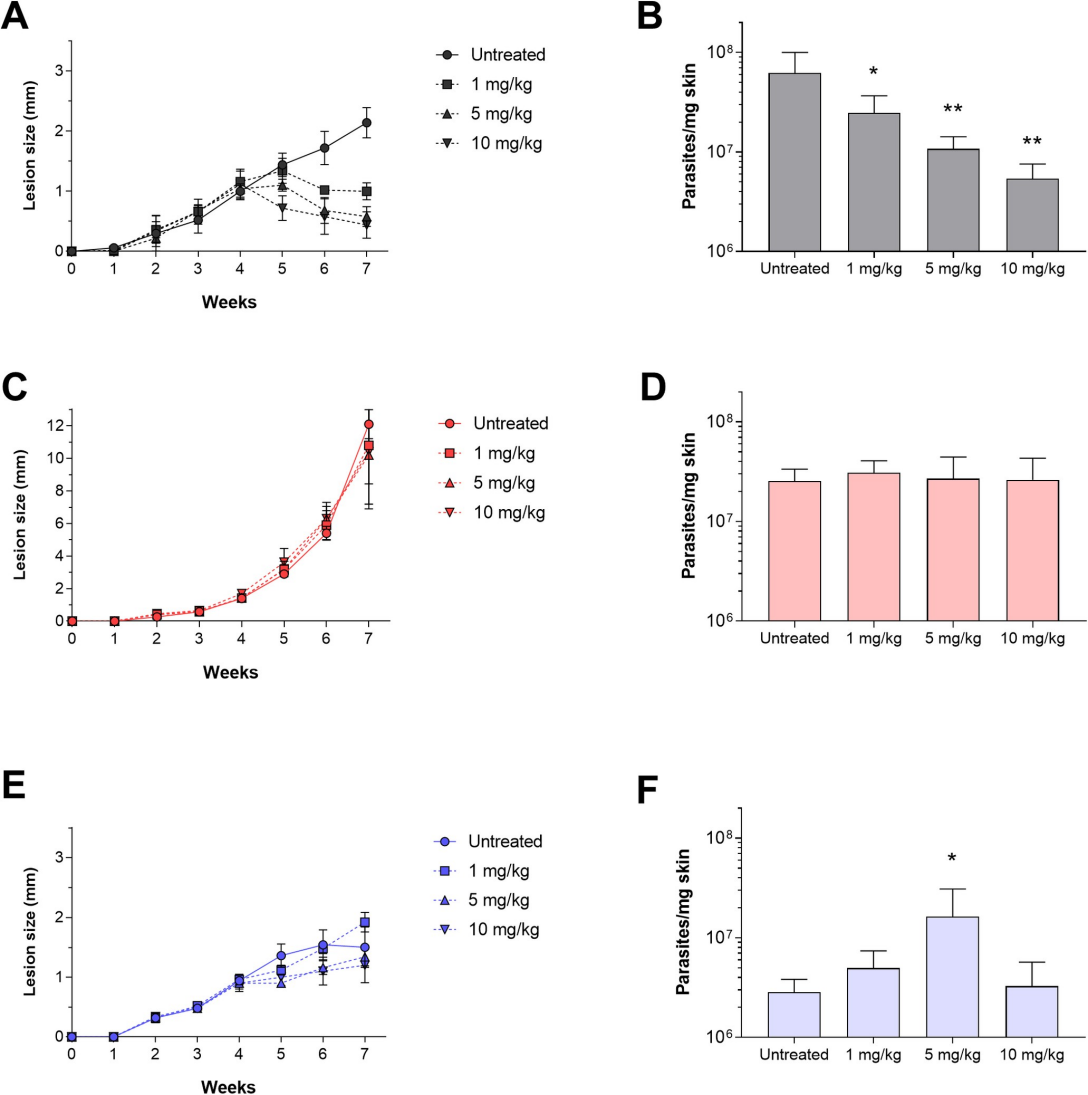
Evaluation of AmB efficacy in mice infected with *L*. *amazonensis* M2269, the AAB clinical isolate, and the AmB200.4 line. Five mice per group were infected with stationary-phase promastigotes of the M2269 strain (A and B), the AAB isolate (C and D), or the AmB200.4 line (E and F). Evolution of lesion size in infected mice over the weeks (A, C, and E). Treatment started on the 4^th^ week post-infection, with dosages of 1, 5, and 10 mg/kg/day of AmB for 15 days. Quantification of parasite burden by quantitative real-time PCR of infected animals at the end of treatment (7^th^ week post-infection) (B, D, and F). Statistical analysis was performed using one-way ANOVA with Tukey’s post-test and statistically significant differences are presented for the AmB-treated groups compared with the respective untreated groups (* *p* <0.05 and ** *p* <0.01).

**Fig 3:**
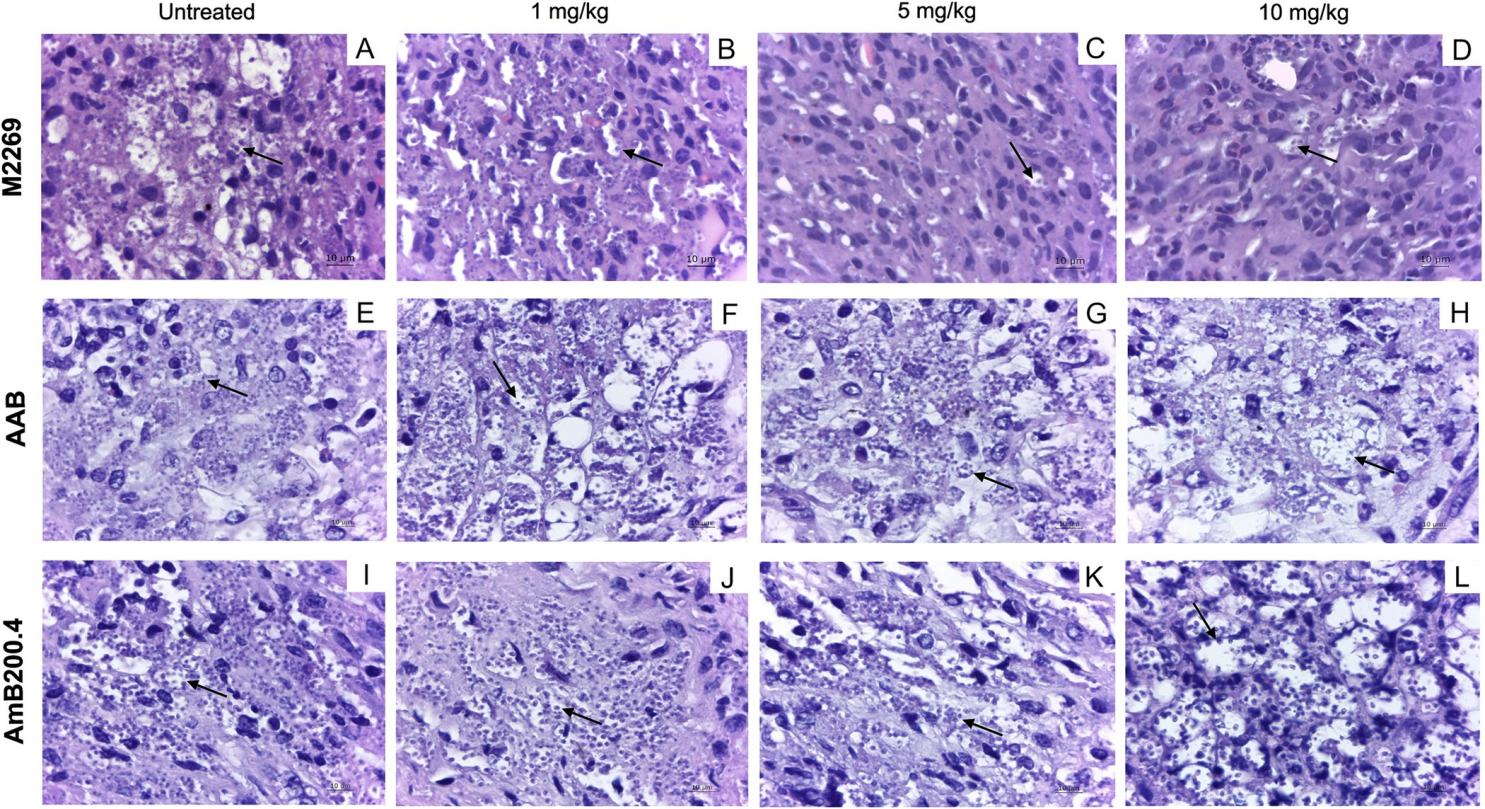
Histological analysis of lesions from mice infected with *L*. *amazonensis* M2269 strain, the AAB clinical isolate, or the AmB-resistant line (AmB200.4). At the end of the treatment with AmB, animals were euthanized and infected hind footpad fragments were collected, washed with PBS, fixed with formalin, and processed with paraffin. Sections were stained with hematoxylin-eosin and visualized on a light microscope. Images of untreated mice and mice treated with 1, 5, or 10 mg/kg/day of AmB for those infected with the M2269 strain (A, B, C, and D, respectively), the AAB isolate (E, F, G, and H, respectively), or the AmB200.4 line (I, J, K, and L, respectively). Arrows indicate amastigotes inside macrophage vacuoles. Bar: 10 μm.

To investigate whether the *in vitro* resistance phenotype observed for the AAB isolate would affect the response to the *in vivo* treatment, we evaluated the efficacy of AmB by intraperitoneal administration in BALB/c mice infected with the clinical isolate, the M2269 strain, and the AmB200.4 line. We found significant differences in the lesion size progression, particularly in animals infected with the AAB isolate, which had larger lesions than those infected with the M2269 strain and the AmB-resistant line after 7 weeks of infection in the untreated groups (Fig 2). Animals infected with the M2269 strain responded to AmB, with lesion size reductions of 55, 80, and 85% for groups treated with 1, 5, and 10 mg/kg/day of AmB, respectively, when compared to the lesion size of the untreated group (Fig 2A). Parasite burden in the lesions reduced in a dose-dependent manner, decreasing by 60, 83, and 91% in mice treated with 1, 5, and 10 mg/kg/day of AmB, respectively (Fig 2B). The effective dose (ED_50_) was determined and corresponded to 0.6 mg/kg of AmB in animals infected with the M2269 strain. On the other hand, infections with the AAB isolate and AmB200.4 line were completely refractory to AmB treatment (Fig 2C and 2E). For both infection groups under all AmB treatment regimens, animals presented an indistinguishable progression of disease and parasite burden as compared with the respective untreated groups (Fig 2C–2F). Additionally, histological analysis of tissues at the site of infection confirmed the reduction of intracellular amastigotes in an AmB dose-dependent manner for mice infected with the M2269 strain (Fig 3A–3D), corroborating the lesion size and parasite burden data. In contrast, the footpads of mice infected with the AAB isolate and AmB200.4 line presented similar numbers of intracellular amastigotes in untreated and treated animals for all AmB dosages (Fig 3E–3L). These findings indicate that the resistant phenotype observed for the clinical isolate and the AmB-resistant line in both stages of the parasite *in vitro* persisted *in vivo*.

We also observed that animals treated with 5 or 10 mg/kg/day of AmB presented piloerection, a sign that can be associated with discomfort, which could perhaps be related to drug toxicity. To assess the possible toxicity of the AmB treatment, the body weight of animals was determined before and post-treatment. No significant difference was observed in the body weight of untreated and AmB-treated groups (S3 Fig). Biochemical tests were also performed to evaluate the renal (creatinine) and hepatic (ALT and AST) function of the animals, and no significant differences were observed in these parameters between the untreated and AmB-treated groups (S4 Fig).

### *In vivo* treatment with MF, PM, and MF plus PM of mice infected with *L*. *amazonensis* AAB clinical isolate

**Fig 4:**
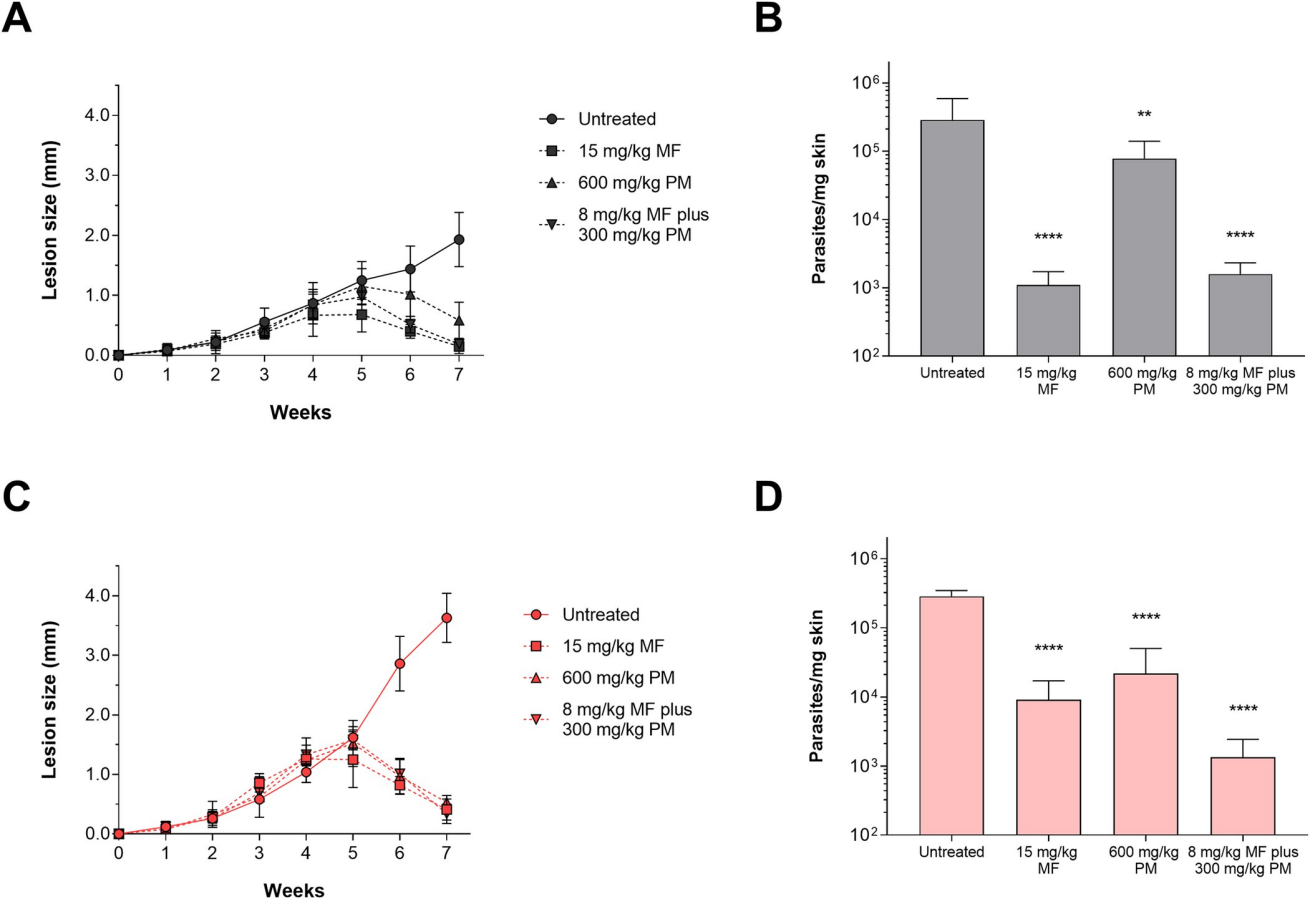
Evaluation of the efficacy of MF and PM monotherapies and combined therapy (MF plus PM) in mice infected with *L*. *amazonensis* M2269 strain or the AAB clinical isolate. Five mice per group were infected with stationary-phase promastigotes of the M2269 strain (A and B) or the AAB isolate (C and D). Evolution of lesion size in infected mice over the weeks (A and C). Treatment started on the 4^th^ week post-infection, with dosages of 15 mg/kg/day of MF, 600 mg/kg/day of PM, or 8 mg/kg/day of MF plus 300 mg/kg/day of PM for 15 days. Quantification of parasite burden by quantitative real-time PCR of mice infected with M2269 strain (B) or the AAB isolate (D) at the end of treatment (7^th^ week post-infection). Statistical analysis was performed using one-way ANOVA with Tukey’s post-test and statistically significant differences are presented for the MF, PM, or combined therapy groups compared with the respective untreated group (** *p* <0.01 and **** *p* <0.0001).

To confirm the *in vivo* AmB resistance phenotype observed for the AAB isolate and exclude the possibility that the non-response to treatment was simply due to higher virulence of this isolate in relation to the M2269 strain, we evaluated the efficacy of MF and PM as monotherapies and in a combination scheme *in vivo*. Although these drugs were not used during the patient’s therapeutic regimen [29], the isolate was considered less susceptible to both drugs when compared with the susceptibility of the M2269 strain in the amastigote stage (Table 1). To evaluate the effectiveness of these drugs, mice were infected with the M2269 strain or the AAB isolate and treated with three different schemes: 15 mg/kg/day of MF, 600 mg/kg/day of PM, or a combined treatment of 8 mg/kg/day of MF plus 300 mg/kg/day of PM. All treatment schemes led to a significant reduction in the lesion size of mice infected with the M2269 strain or AAB isolate (Fig 4A and 4C); on average, lesion size reduced by 92, 69, and 90% respectively, in mice infected with the M2269 strain and 78, 72, and 82% respectively, in mice infected with the AAB isolate as compared to the respective untreated groups (Fig 4A and 4C). Groups treated with MF, PM, or MF plus PM presented a significant reduction in parasite burden when compared with the respective untreated groups (Fig 4B and 4D). The combined therapy showed at least a 100-fold reduction in parasite load in mice infected with the M2269 strain or AAB isolate (Fig 4B and 4D). On the other hand, PM monotherapy resulted in around a 10-fold reduction in parasite burden when compared with the respective untreated groups. Finally, animals treated with MF presented a significant reduction in parasite burden, as animals infected with the M2269 strain presented a reduction of at least 100-fold, while those infected with the AAB isolate had a reduction of approximately 30-fold (Fig 4B and 4D).

## Discussion

The main goal of this study was to characterize the *in vitro* and *in vivo* drug susceptibility of a *L*. *amazonensis* isolate obtained from a DCL patient coinfected with HIV who had been exposed to a set of antileishmanial drug treatment schemes, including SbV, L-AmB, and PEN, with no effective response [29]. To evaluate whether the treatment failure could be related to parasite drug resistance, we tested the susceptibility of the isolate to the main drugs used in the chemotherapy of leishmaniasis. Our findings showed that this isolate presented low *in vitro* susceptibility in the amastigote stage to two drugs used in the treatment of the patient, SbV (Glucantime) and L-AmB. The reported clinical cure rates of SbV for patients with TL are below 50% [5,6,38], even though they are still the main drug used in the chemotherapy of leishmaniasis in Brazil [4]. AmB has been considered as the leading drug to treat leishmaniasis, particularly in its less toxic formulation, L-AmB; this can be explained in part by its high efficacy in the treatment of TL, with cure rates that vary from 88 to 97% [4,39,40]. Furthermore, this isolate was uniformly susceptible to PEN, which had been used in the patient treatment, while for MF and PM, drugs to which the patient had not been exposed, low *in vitro* susceptibility was found when compared with that of the reference strain M2269, which is considered susceptible to all these drugs [33,34].

Considering that previous studies on *in vitro* drug susceptibility have shown that *L*. *amazonensis* Brazilian clinical isolates are uniformly susceptible to AmB [36,37] and that AmB resistance has not been reported in Brazilian endemic areas for patients treated with this drug, we focused our investigation on the AmB resistance phenotype observed for the AAB isolate. The resistance phenotype was evaluated in parallel with an experimentally selected AmB-resistant line, generated using the *L*. *amazonensis* M2269 as the parental strain, and a similar profile of *in vitro* resistance was found when compared with that of the AAB isolate, particularly at the amastigote stage. The AmB200.4 line showed a slower growth rate when compared to that of the parental M2269 strain, while the growth rate of the isolate was similar to that of the reference strain. Experimentally generated AmB-resistant parasites of *L*. *donovani* and *L*. *mexicana* reportedly had similar growth rates when compared with those of the corresponding parental strains [24,41]. In the amastigote stage, the AAB isolate was able to infect more macrophages than the M2269 strain and AmB200.4 line; while more amastigotes per infected macrophage were observed for the AAB isolate and AmB-resistant line in absence of drug. Differently, a *L*. *donovani* AmB-resistant line showed reduced infectivity when compared to that of the parental strain, and a reduction of the sterol levels at the plasma membrane was hypothesized as responsible for this reduced infectivity [41,42].

Although reports on *Leishmania* isolates with AmB resistance are scarce in the literature, with none reported for TL-causative species, AmB resistance can be easily generated in the laboratory. In *Leishmania* spp., the AmB resistance phenotype has mainly been associated with the loss of AmB-binding ergosterol via mutations and/or structural alterations in genes involved in ergosterol biosynthesis [24,26–28,43]. In *L*. *donovani*, an AmB-resistant line (experimentally selected in the promastigote stage) showed alterations in the membrane lipid composition, with a prevalence of saturated fatty acids, and the main sterol was an ergosterol precursor, cholesta-5,7,24-trien-3β-ol, not ergosterol, as is observed in AmB-susceptible parasites and considered the main sterol in *Leishmania* [25]. Additionally, AmB resistance may induce changes in membrane fluidity, affecting the affinity of AmB with the plasma membrane of the parasite [20,25]. Although the molecular basis of AmB resistance in the AmB200.4 line and the AAB isolate was not investigated, we speculate that similar mechanisms may be involved, especially for the AmB200.4 line that was selected in a similar way to that described in the abovementioned studies. The AmB-resistant line was also resistant to MF, with an EC_50_ almost 4-fold higher than that for the M2269 strain in the amastigote stage. Interestingly, in some AmB-resistant parasites selected *in vitro*, mutations or deletions of the P-type ATPase transporter gene, also known as MF transporter, which is associated with MF resistance, have been found, indicating that this *locus* may be involved in the resistance to both drugs in *Leishmania* [24,44,45].

Owing to the low *in vitro* susceptibility of the isolate to AmB, the *in vivo* AmB efficacy in infected BALB/c mice was investigated. The AmB200.4 line was used to evaluate and compare the effectiveness of AmB. For animals infected with the M2269 strain, a significant reduction in lesion size and parasite burden was observed in treated groups compared with that of the untreated animals. A dose-dependent treatment effect was observed and animals treated with the highest AmB dosage (10 mg/kg/day) exhibited a decrease in the number of parasites per mg of lesion by more than 10-fold in relation to the untreated group. Similar findings were described by [46], who evaluated the *in vivo* efficacy of AmB in mice infected with the M2269 strain treated with dosages of 1.2, 2, and 4 mg/kg/day over 20 days. The ED_50_ values, based on limited dilution and luciferase assays of parasite burden, were 1.19 to 1.89 mg/kg/day, respectively. Here, the ED_50_ value was 0.6 mg/kg/day and the quantification of parasite burden was determined by quantitative real-time PCR. Despite differences between these methods, the ED_50_ values were similar and a reduction in the parasite burden in a dose-dependent manner was observed. Other studies have also shown that AmB was able to significantly reduce parasite burden in mice infected with different species responsible for TL and VL, such as *L*. *major*, *L*. *mexicana*, and *L*. *donovani* [24,47,48].

On the other hand, mice infected with the clinical isolate or the AmB200.4 line and treated with AmB showed no reduction in lesion size and parasite burden in any of the treated groups, and thus, did not respond to the *in vivo* treatment. To our knowledge, this is the first report of AmB clinical resistance, experimentally confirmed *in vitro* and *in vivo*, caused by a Brazilian *L*. *amazonensis* clinical isolate. It is important to state that lesions in mice caused by infection with the AAB isolate were significantly larger when compared with those caused by the M2269 strain and AmB200.4 line. Variations in lesion size and parasite burden have previously been reported in BALB/c mice infected with two distinct *L*. *amazonensis* strains [49].

A *L*. *donovani* isolate from an AmB-unresponsive VL patient was characterized *in vitro*, which revealed an EC_50_ at least 8-fold higher than that of sensitive parasites; the isolate exhibited changes in the membrane composition and upregulation in the thiol metabolic pathway, leading to reduced AmB accumulation [20]. In that study, AmB clinical resistance was not experimentally confirmed *in vivo*. Differently, the *in vitro* susceptibility of clinical isolates obtained from HIV patients coinfected with *L*. *infantum* that had undergone several courses of AmB treatment were uniformly susceptible to AmB [16]. Indeed, several cases of AmB treatment failure have been reported in cases of immunosuppression owing to HIV coinfection or not, but not directly associated with drug resistance [15,50–52]. These findings indicate that AmB resistance is still rare in the field, probably owing to that fact that this drug is not widely used, particularly in Brazil, where it is still considered expensive. AmB is generally used as a second or third option after treatment failure, as described for the patient infected with the AAB isolate [29] that, as we report here, presented an *in vitro* and *in vivo* resistance phenotype. The expansion of AmB use as a monotherapy may favor the selection of resistant parasites in endemic areas, limiting its use in the treatment of the disease.

In *in vivo* assays, toxicity signs potentially caused by the AmB treatment were investigated. Our data showed that none of the dosages of AmB used affected the body weight of animals. Beyond that, we evaluated liver and renal toxicity through the serum levels of AST, ALT, and creatinine. No significant changes were observed between groups treated with the administered dosages of AmB. Recently, AmB deoxycholate and three of its derivatives (AmBisome, AmB amino-urea, and AmB methyl-urea) reportedly did not cause renal toxicity in animals infected with *L*. *donovani* [47]. However, animals treated with AmB amino-urea at concentrations of 4 and 8 mg/kg/day exhibited liver toxicity, with increased serum levels of ALT [47]. The effectiveness of two L-AmB formulations (Fungisome and AmBisome) in mice infected with *L*. *major* and treated with intravenous dosages of 5, 10, and 15 mg/kg/day for 10 days were evaluated [48]. A reduction in parasite load was observed in animals treated with 5 and 10 mg/kg/day of Fungisome, with no significant differences in parasite burdens when compared with those of animals treated with the same doses of AmBisome [48]. However, 15 mg/kg/day of Fungisome was toxic, leading to death; while animals treated with the same dosage of AmBisome remained alive, demonstrating that this formulation is less toxic in mice [48]. In fact, both formulations are considered less toxic and better tolerated than AmB deoxycholate [4].

Owing to the increase in patient unresponsiveness to current antileishmanial treatment regimens, the evaluation of alternative therapeutic schemes is necessary. While MF was only recently approved in Brazil for the treatment of TL, PM is used mainly for the treatment of VL in combination with SbV or MF, with a good clinical response [10,53]. Although the clinical isolate in the amastigote stage was less susceptible to MF and PM when compared with the susceptibility observed for the M2269 strain, the *in vivo* treatment response of animals infected the AAB isolate responded to all treatment schemes (MF and PM as a monotherapy and in combination), with sustained reduction in lesion size and parasite burden in treated AAB-infected groups. The combination of MF plus PM was the most effective, with a significant reduction of lesion size and parasite burden for animals infected with the AAB isolate when compared to those of animals treated with the MF and PM monotherapies. In addition, the drug combination was also effective in animals infected with the M2269 strain, with a similar reduction in lesion size and parasite burden as animals treated with the MF monotherapy. Interestingly, the combined treatment of MF plus PM (20 mg/kg/day and 350 mg/kg/day respectively for 5 days) in hamsters infected with *L*. *infantum* showed a cumulative efficacy, with reduction in parasite burden >98% in liver, spleen, and bone marrow, when compared with animals treated with the respective monotherapies that presented reductions between 74.4 and 94.5% [54]. Previously, the efficacy of MF against a *L*. *amazonensis* clinical isolate, obtained from a DCL patient, was evaluated as a therapeutic option in this case. As in this study, the isolate was less susceptible *in vitro* to MF but responded to treatment *in vivo* [33]. Differently to what was observed for AmB, no correlation was observed in the *in vitro* susceptibility and *in vivo* treatment response to MF for the AAB isolate. Animals infected with the M2269 strain or the AAB isolate and treated with PM presented a similar reduction in lesion size and parasite burden, despite a lower *in vitro* susceptibility of the AAB isolate in the amastigote stage. Finally, PM was described to be more effective using the same *in vivo* model infected with a clinical isolate highly susceptible *in vitro* when compared with the M2269 strain that was at least 100-fold more resistant in intracellular amastigotes [34].

In conclusion, our findings showed that AmB was ineffective *in vivo* against a *L*. *amazonensis* clinical isolate (obtained from a DCL patient coinfected with HIV), which also presented an *in vitro* AmB-resistant phenotype, experimentally confirming the clinical resistance to AmB in this patient. The resistance phenotype was specifically associated with AmB, as this isolate responded *in vivo* to different treatment schemes involving drugs that had not been used in the treatment of the patient. The molecular basis responsible for the AmB resistance phenotype is still undetermined for this isolate. Finally, our findings highlight the need of monitoring the drug susceptibility of isolates, as this may be useful for detecting resistant parasites in endemic areas and providing treatment alternatives for patients with leishmaniasis.

## Supporting information

S1 Fig Schematic representation of *in vitro* selection of AmB-resistant parasites through stepwise selection. Each passage of the parasite population in culture is indicated by black circles.
(TIF)

S2 Fig Promastigote growth profile of the M2269 strain, the AAB isolate, and the AmB-resistant line (AmB200.4). Parasites (2×10^5^/mL) were cultivated at 25°C in M199 medium and the number of parasites was determined daily for 7 days.
(TIF)

S3 Fig Average body weight in grams (g) of untreated and AmB-treated groups (15 animals per group) before and post-treatment. Statistical analysis was performed using one-way ANOVA followed by Tukey’s post-test and no statistically significant differences were observed between groups.
(TIF)

S4 Fig Biochemical parameters of mice after treatment with AmB. Serum levels of (A) ALT, (B) AST, and (C) creatinine of untreated and AmB-treated mice (1, 5, or 10 mg/kg/day for 15 days) were analyzed and compared with those of the untreated group. The mean values ± standard deviation of three mice per group is shown. Statistical analysis was performed using one-way ANOVA with Tukey’s post-test and no statistically significant differences were observed between groups.
(TIF)

S1 Table *In vitro* activity of AmB against AmB-resistant parasites. AmB susceptibility of the *L*. *amazonensis* wild-type strain (M2269) and four clones of the AmB-resistant population (selected up to 200 nM of AmB through stepwise selection).
(PDF)
