# Supplementary figures and images for: Amphotericin B resistance in Leishmania amazonensis: In vitro and in vivo characterization of a Brazilian clinical isolate
Source: PLoS Negl Trop Dis. 2024 May 20;18(5):e0012175. doi: 10.1371/journal.pntd.0012175 (PMC11142706; doi:10.1371/journal.pntd.0012175)

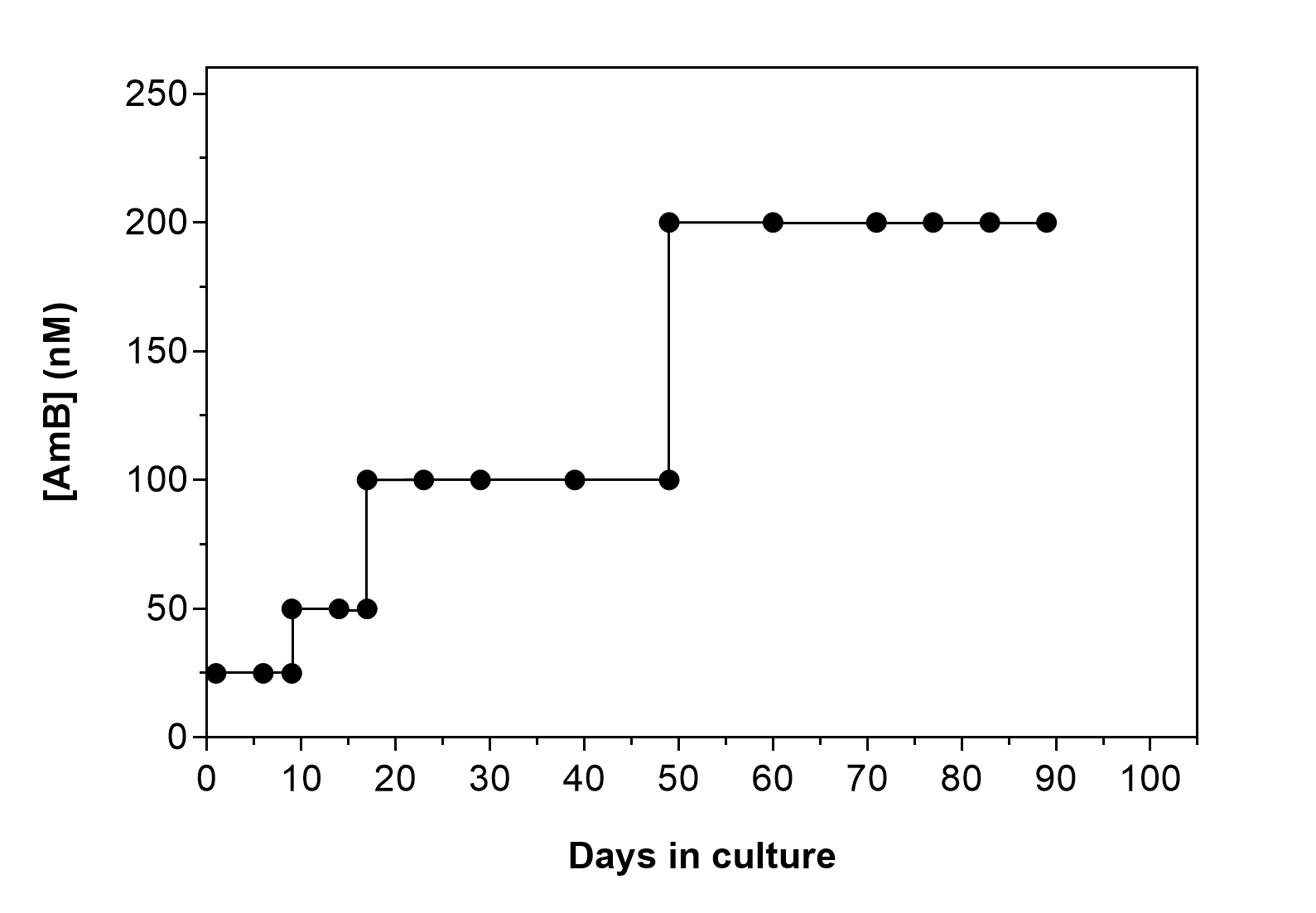

Supplement: S1 Fig — Each passage of the parasite population in culture is indicated by black circles. (TIF) [file pntd.0012175.s001.tif]

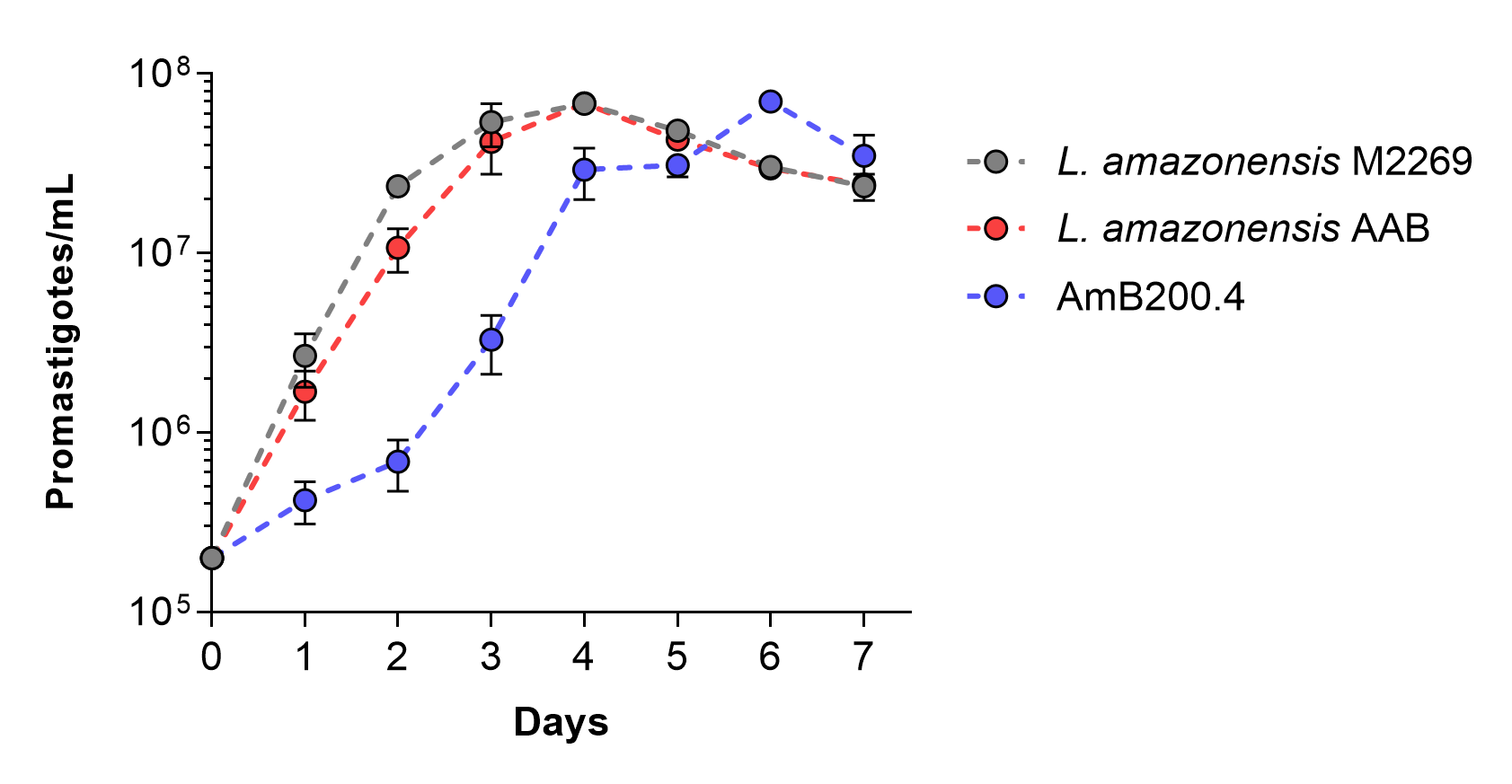

Supplement: S2 Fig — Parasites (2×10^5^/mL) were cultivated at 25°C in M199 medium and the number of parasites was determined daily for 7 days. (TIF) [file pntd.0012175.s002.tif]

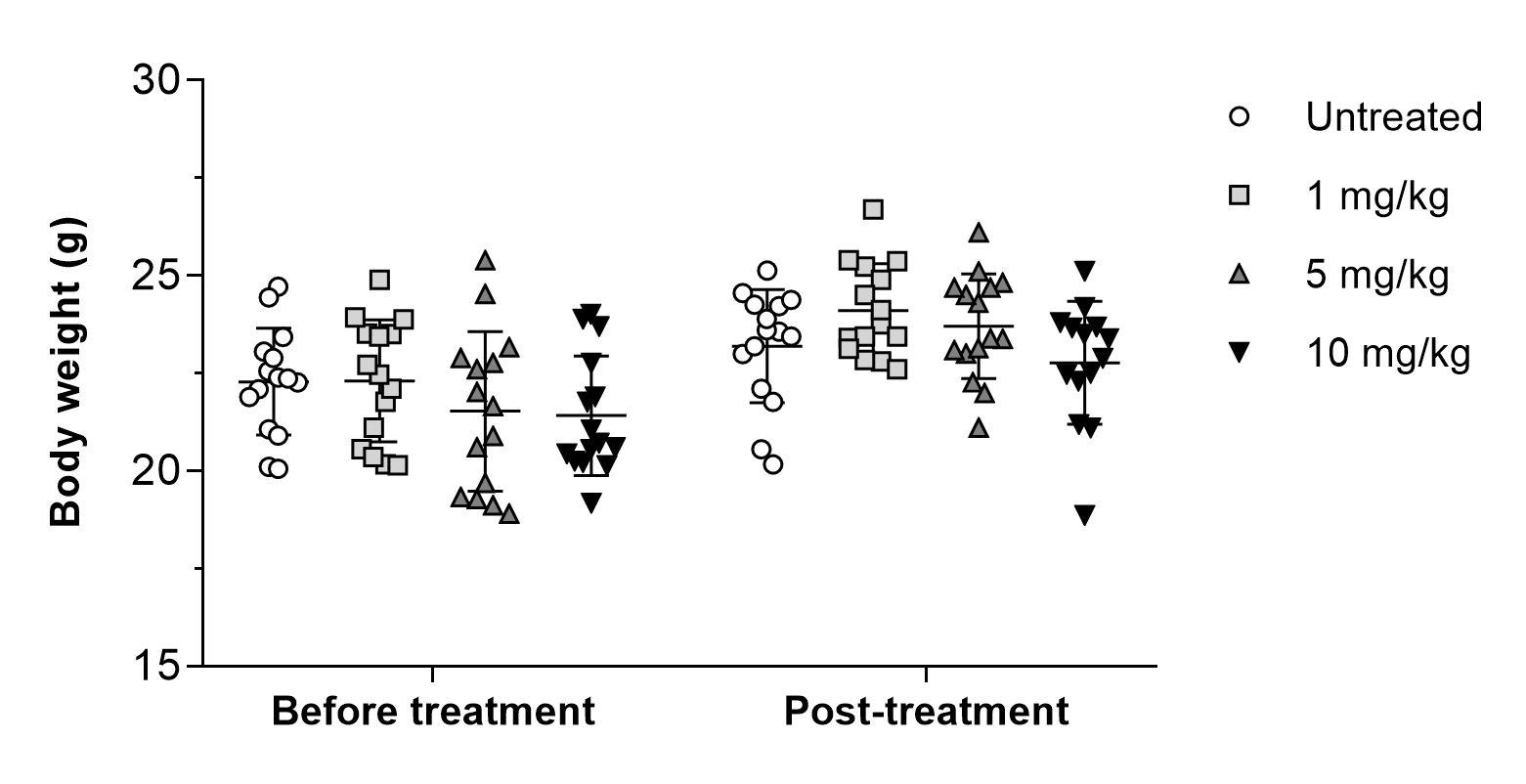

Supplement: S3 Fig — Statistical analysis was performed using one-way ANOVA followed by Tukey’s post-test and no statistically significant differences were observed between groups. (TIF) [file pntd.0012175.s003.tif]

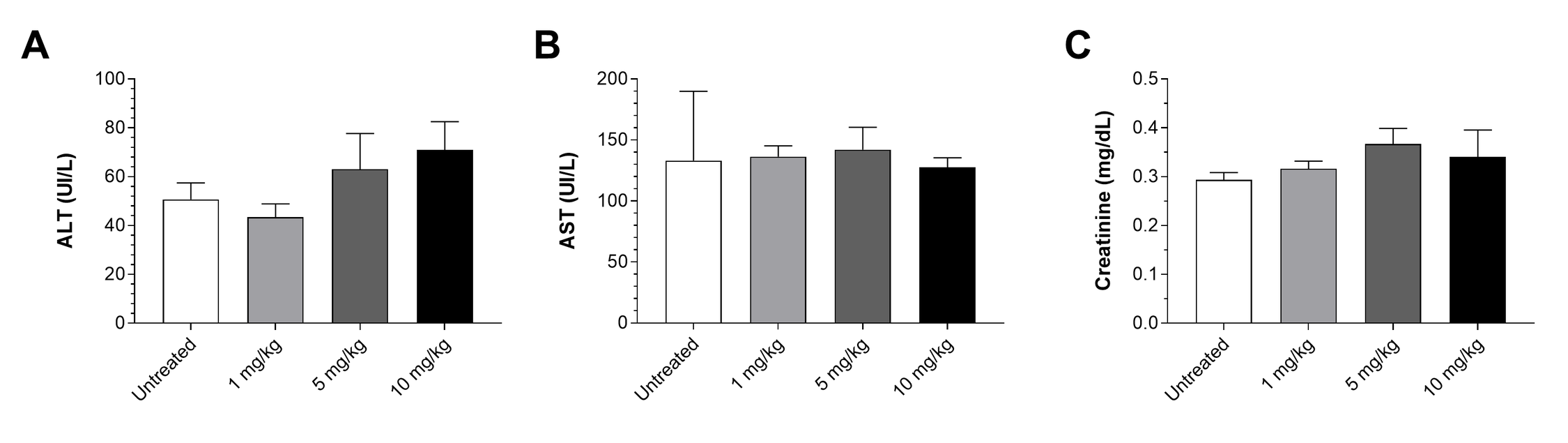

Supplement: S4 Fig — Serum levels of (A) ALT, (B) AST, and (C) creatinine of untreated and AmB-treated mice (1, 5, or 10 mg/kg/day for 15 days) were analyzed and compared with those of the untreated group. The mean values ± standard deviation of three mice per group is shown. Statistical analysis was performed using one-way ANOVA with Tukey’s post-test and no statistically significant differences were observed between groups. (TIF) [file pntd.0012175.s004.tif]
