# Supplementary material for: Amphotericin B resistance in Leishmania amazonensis: In vitro and in vivo characterization of a Brazilian clinical isolate
Source: PLoS Negl Trop Dis. 2024 May 20;18(5):e0012175. doi: 10.1371/journal.pntd.0012175 (PMC11142706; doi:10.1371/journal.pntd.0012175)
Supplement: S1 Table — AmB susceptibility of the L. amazonensis wild-type strain (M2269) and four clones of the AmB-resistant population (selected up to 200 nM of AmB through stepwise selection). (PDF) [file pntd.0012175.s005.pdf]

S1 Table. *In vitro* activity of AmB against AmB-resistant parasites. AmB susceptibility of the *L. amazonensis* wild-type strain (M2269) and four clones of the AmB-resistant population (selected up to 200 nM of AmB through stepwise selection).

**Strain/ Resistant line**	**Promastigote**	
	**EC_{50} [1]**	**(n) [2]**
M2269 (WT)	29.02 ± 4.46	3
AmB200 (population)	183 ± 4.37	3
AmB200.1	122.7 ± 7.56	3
AmB200.2	135.7 ± 13.70	3
AmB200.3	81.15 ± 2.58	3
AmB200.4	124.73 ± 3.78	3

[1] EC_{50} mean values ± standard deviation in nM;
[2] Number of independent experiments.
